# Analysis of genetic variation and diversity of Rice stripe virus populations through high-throughput sequencing

**DOI:** 10.3389/fpls.2015.00176

**Published:** 2015-03-24

**Authors:** Lingzhe Huang, Zefeng Li, Jianxiang Wu, Yi Xu, Xiuling Yang, Longjiang Fan, Rongxiang Fang, Xueping Zhou

**Affiliations:** ^1^State Key Laboratory of Rice Biology, Institute of Biotechnology, Zhejiang University, HangzhouPeople’s Republic of China; ^2^Institute of Crop Science and Institute of Bioinformatics, Zhejiang University, HangzhouPeople’s Republic of China; ^3^State Key Laboratory of Plant Genomics, Institute of Microbiology, Chinese Academy of Sciences, BeijingPeople’s Republic of China

**Keywords:** Rice strip virus, population, diversity, deep sequencing

## Abstract

Plant RNA viruses often generate diverse populations in their host plants through error-prone replication and recombination. Recent studies on the genetic diversity of plant RNA viruses in various host plants have provided valuable information about RNA virus evolution and emergence of new diseases caused by RNA viruses. We analyzed and compared the genetic diversity of Rice stripe virus (RSV) populations in *Oryza sativa* (a natural host of RSV) and compared it with that of the RSV populations generated in an infection of *Nicotiana benthamiana*, an experimental host of RSV, using the high-throughput sequencing technology. From infected *O. sativa* and* N. benthamiana* plants, a total of 341 and 1675 site substitutions were identified in the RSV genome, respectively, and the average substitution ratio in these sites was 1.47 and 7.05 %, respectively, indicating that the RSV populations from infected *N. benthamiana* plant are more diverse than those from infected *O. sativa* plant. Our result gives a direct evidence that virus might allow higher genetic diversity for host adaptation.

## Introduction

RNA viruses are known for their high evolutionary potential due mainly to their error-prone replication, and fast rate of genome replication (Domingo and Holland, 1997; Roossinck, 1997; Elena and Sanjuán, 2007; Lauring and Andino, 2010; Sanjuan et al., 2010). In addition, template-switching of viral RNA-dependent RNA polymerase (RdRp) during virus replication, known as RNA recombination, also functions to accelerate virus evolution (Nagy and Simon, 1997; Garcia-Arenal et al., 2001). Using these strategies, viruses are capable of bringing beneficial mutations into and removing deleterious mutations from their genomes (Worobey and Holmes, 1999). Mutation in the viral genomic sequence may lead to changes of virus host range, disease symptoms, and emergence of new viruses in nature. Previous reports have indicated that mutant viruses often appeared soon after the RNA virus infected its host plant and to form diverse populations in infected cells, and these studies were analyzed through sequencing random selected clones derived from an infected plant (Domingo and Holland, 1997; Roossinck, 1997; Schneider and Roossinck, 2000; Garcia-Arenal et al., 2001, 2003).

Rice stripe virus (RSV) is one of the most damaging rice pathogens in China and was firstly identified in rice field in China in 1963 (Lin et al., 1990). It is now identified in rice fields in 16 provinces of China and caused significant yield losses (Xiong et al., 2008; Wei et al., 2009). RSV is the type member of the genus *Tenuivirus* and infects mainly rice and a few other species in the family *Poaceae* including maize, oat and wheat. In field, RSV is transmitted efficiently by small brown plant hopper (*Laodelphax striatellus*) in a persistent manner. RSV can propagate within the insect vector and transmitted to its progenies (Koganezawa et al., 1975; Falk and Tsai, 1998). Under the laboratory conditions, RSV can be transmitted to an experimental host, *Nicotiana benthamiana,* through mechanical inoculation (Kong et al., 2014).

**FIGURE 1:**
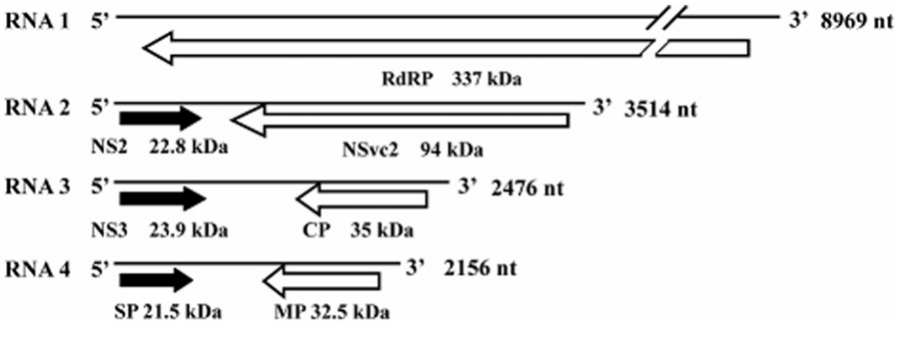
**Genome organization of Rice stripe virus (RSV)**. ORFs are indicated as black arrows on the viral-sense RNAs and white arrows on the viral complementary-sense RNAs. Direction of arrowheads indicates the direction of translation. RdRp, RNA-dependent RNA polymerase; CP, coat protein; SP, disease-specific protein; MP, movement protein; NS2 and NS3, non-structural proteins on the viral-sense RNA 2 and 3; NSvc2, non-structural protein on the viral complementary-sense RNA2.

Rice stripe virus is a single-stranded RNA virus containing four genome segments, designated as RNA 1, 2, 3, and 4 (**Figure 1**; Zhu et al., 1991, 1992; Takahashi et al., 1993; Toriyama et al., 1994). RNA 1 is in negative sense and encodes a putative protein of approximately 337 kDa. This protein was suggested to be part of the RSV RdRp complex and is associated with the RSV filamentous ribonucleoprotein (Toriyama et al., 1994). The other three RNA segments of RSV are all ambisense and each RNA segment contains two ORFs, one is in the viral sense (vRNA) and the other in the viral complementary-sense (vcRNA; Ramirez and Haenni, 1994). The vRNA 2 ORF encodes a membrane-associated protein of 22.8 kDa and the vcRNA 2 ORF encodes a poly-glycoprotein of 94 kDa. Functions of these proteins are still unknown (Takahashi et al., 1993; Falk and Tsai, 1998). The vRNA 3 ORF encodes a 23.9-kDa suppressor of RNA silencing (Xiong et al., 2009) and the vcRNA 3 ORF encodes a nucleocapsid protein of 35 kDa (Kakutani et al., 1991; Zhu et al., 1991). The vRNA 4 ORF encodes a major non-capsid protein of 21.5 kDa that functions in disease symptom development (Kong et al., 2014). The vcRNA 4 ORF encodes a protein of 32.5 kDa and is required for RSV cell-to-cell movement in infected plant (Xiong et al., 2008).

Determination of the complexity of RNA virus population in their host plants requires a rapid, reliable, and cost-effective sequencing method. Because viral RNAs with specific mutations may accumulate to lower levels than the most frequent viral RNAs in infected cells, traditional small-scale sequencing used in many earlier studies might fail to detect these non-conserved or lowly accumulated viral RNAs. Although the evolution of RSV over long periods of time and in areas covering large geographical districts in China has been studied (Wei et al., 2009), the population diversity of RSV within a plant is still unknown. In this study, we used the high-throughput sequencing technology (also known as the second generation sequencing technology) to investigate the diverse population of RSV in an infected *Oryza sativa* plant (a natural host) and compare it with that observed in an infected *N. benthamiana* plant (an experimental host) and we found that the RSV populations from the infected *N. benthamiana* plant are more diverse than those from the infected *O. sativa* plant. Our results give a direct evidence that virus might allow higher genetic diversity for host adaptation.

## Materials and Methods

### Virus Source, Host Plants, and Virus Inoculation

Infected *O. sativa* plants with typical RSV symptoms were collected from rice fields in Jiangsu Province, China. A single plant with only RSV infection was identified through RT-PCR using specific primers and ELISA using specific antibodies as described previously (Wang et al., 2004) and leaves of this plant were then stored at -80°C till use. *N. benthamiana* seedlings were grown inside an insect-free room with a constant temperature at 25°C and a 16 h light supply. Leaves from the RSV-infected *O. sativa* plant were ground in 0.1 M phosphate buffer, pH 7.0, and the crude extract was mechanically inoculated to leaves of *N. benthamiana* seedlings at six- to seven-leaf stages. Young systemic leaves of one *N. benthamiana* plant showing yellow vein symptoms were harvested at 30 days post inoculation (dpi). The harvested fresh *N. benthamiana* leaves and the frozen RSV-infected *O. sativa* leaves were used for the viral population analysis.

### Viral cDNA Library Construction and Sequencing

Total RNA was extracted from 1g leaf tissues of the frozen *O. sativa* and fresh *N. benthamiana* using TRIzol reagent following manufacturer’s instructions (Invitrogen, Carlsbad, CA, USA), respectively, and cDNA libraries of RSV were then constructed. First strand cDNA was synthesized using the SuperScript III reverse transcriptase (Invitrogen, Carlsbad, CA, USA), primers used for first strand cDNA synthesis were designed according to the highly conserved 3′ terminal regions of the four RSV RNAs: 5′-acacaaagtccagaggaaaacaa-3′ for RNA 1, 5′-acacaaagtctgggtataacttctt-3′ for RNA 2, 5′-acacaaagtcctgggtaaaatag-3′ for RNA 3, and 5′-acacaaagtccagggcatttgt-3′ for RNA 4. Second strand cDNAs were synthesized using DNA polymerase I and RNase H. Ends of double strand cDNAs were repaired using T4 DNA polymerase and Klenow DNA polymerase. cDNA pair-end libraries were prepared using standard Illumina protocols and then the libraries were sequenced using Illumina Genome Analyzer II instrument (Illumina, San Diego, CA, USA).

### Bioinformatics Analysis

To minimize artificial mistakes from sequencing errors, short reads were pre-processed using the FASTX-Toolkit (http://hannonlab.cshl.edu/fastx_toolkit/) by removing the low quality reads (below Q30) and adaptors. The reads representing each sample were mapped onto the reference sequences of RSV genome segments (NC_003753.1, NC_003754.1, NC_003755.1, and NC_003776.1) using the MAQ program (Mapping and Assembly with Qualities, Version 0.7.1) with default parameters (Li et al., 2008).

## Results

### High-throughput Sequencing of RSV Genome from Infected *O. sativa* and *N. benthamiana* Plants

The cDNA libraries derived from RSV-infected *O. sativa* plant and that prepared from RSV-infected *N. benthamiana* plant were sequenced, using the Illumina/Solexa platform. The sequences have been submitted to Sequence Read Archive (SRA) with accession number SRP051574. A total of 27,965,556 reads were obtained and 6.67% of them were mapped to the RSV genomic sequence. The average sequencing depths of the RSV genome segments in *O. sativa* were from 953.8 (RNA2) to 2129.1 (RNA4) and those in *N. benthamiana* were from 3260.2 (RNA1) to 7330.5 (RNA3). The number of mapped bases and the sequencing depth of each RSV RNA segment are shown in **Table 1**. Our results indicate that the data we obtained are sufficient to represent the RSV populations in a single infected plant.

**Table 1:** Number of mapped bases and sequencing depth of RSV genome segments.

Genome segment	Mapped bases		Sequencing depth	
	*Oryza sativa*	*Nicotiana benthamiana*	*Oryza sativa*	*Nicotiana benthamiana*
RNA1	10374012	29240682	1156.7	3260.2
RNA2	3351675	25596930	953.8	7284.3
RNA3	7833087	18150381	3163.6	7330.5
RNA4	4590294	11924373	2129.1	5530.8

### Genetic Diversity of RSV Populations in *O. sativa*

**FIGURE 2:**
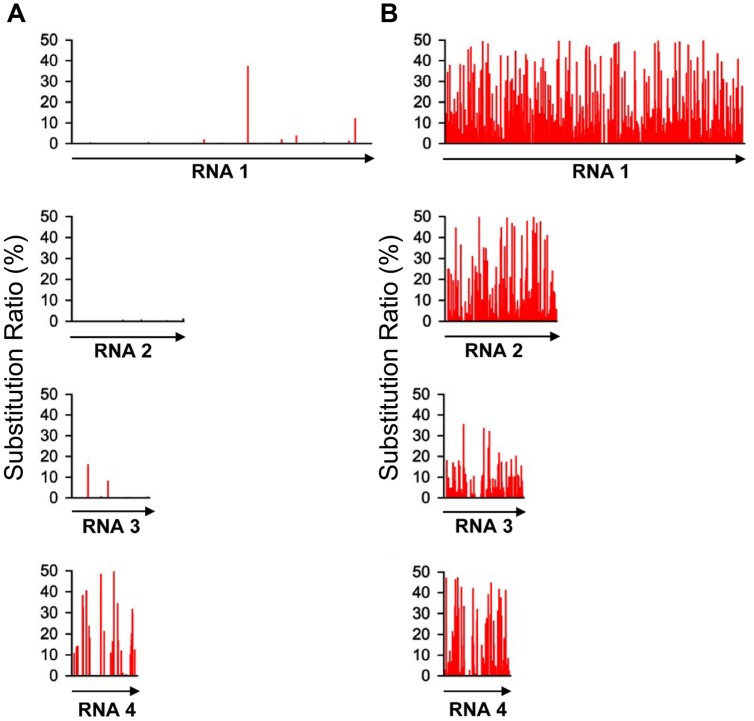
**Substitution patterns in the RSV genome occurred in *Oryza sativa* (A) and *Nicotiana benthamiana* (B)**. The substitution pattern in each of the four RSV RNA segments is presented. The vertical coordinates represent the substitution ratios derived from the master sequence of each populations at each site (number of substituted nucleotide observed/number of total nucleotide sequenced).

**FIGURE 3:**
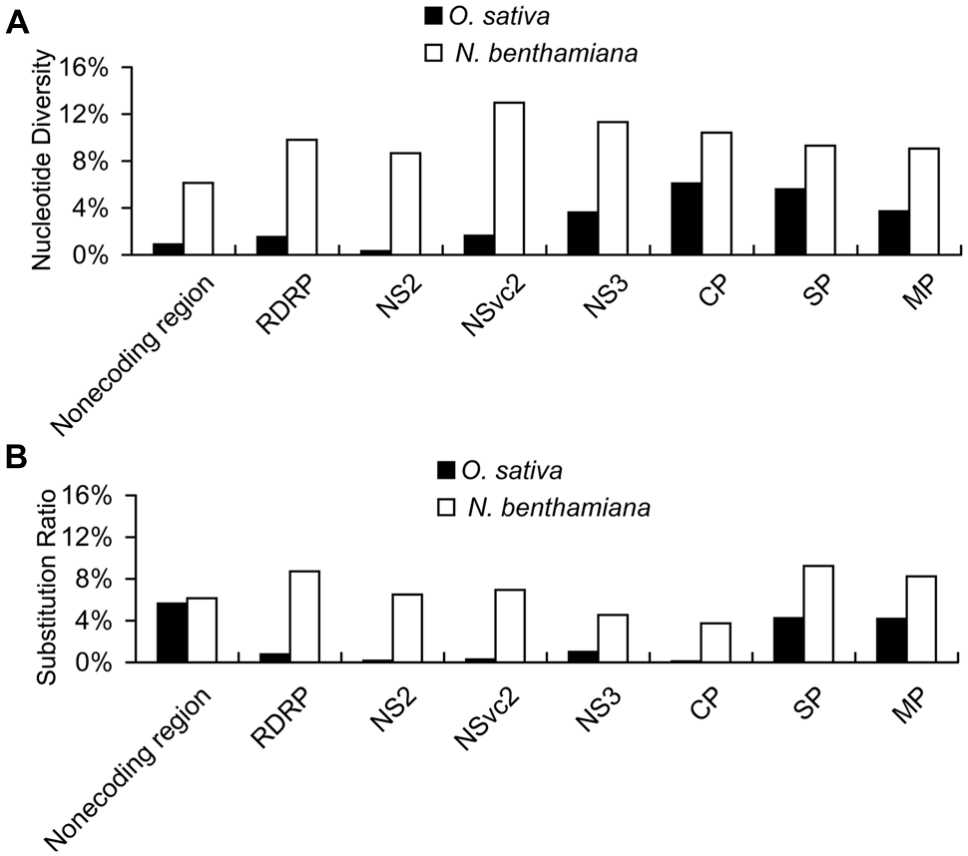
**Average nucleotide diversity (A) and average substitution ratio (B) of each ORF and non-coding regions in RSV quasispecies from *O. sativa* and *N. benthamiana.*** Average nucleotide diversity represents number of sites with substitution/number of total sites of corresponding sequence and substitution ratio represents number of substituted nucleotide observed/number of total nucleotide sequenced.

With the high-throughput sequencing results, a “two-dimensional view” of RSV populations is drawn. This includes average nucleotide diversity (number of sites with substitution/number of total sites of corresponding sequence) and substitution ratio within each site (number of substituted nucleotide observed/number of total nucleotide sequenced). Substitutions determined in the RSV populations from the infected *O. sativa* plant located at 123 different sites in RNA 1 (1.37%), 22 sites in RNA 2 (0.63%), 76 sites in RNA 3 (3.07%), and 60 sites in RNA 4 (2.78%). Among the four RSV RNAs, substitutions occurred more densely in RNA 4. There are 13 sites with substitution ratio above 20% in RSV RNAs and all of them were found in RNA 4, except one at the 3570th site in RNA 1 (**Figure 2A**). Average nucleotide diversities in the *NS3*, *CP*,* SP*, and *MP* ORFs were found to be much higher than those in the *RdRp, NS2, NSvc2* ORFs and non-coding regions (**Figure 3A**). Although the average nucleotide diversities in the *NS3* and *CP* ORFs were similar to those in the *SP* and *MP* ORFs, the average substitution ratios in these two ORFs were much lower (**Figure 3B**). Interestingly, the average substitution ratio (5.64%) in non-coding regions was higher than any ratios found in the ORFs analyzed. The average nucleotide diversity in non-coding regions was, however, only 0.89%, lower than all ORFs analyzed except the *NS2* (**Figure 3**).

Transitions (i.e., A↔G and C↔T) were detected at 238 sites and transversions (i.e., A ↔ C, A ↔ T, G ↔ C, and G ↔ T) were found at 103 sites. The ratio of transition to transversion was about 2.3 (**Table 2**). A previous report also indicated that transitions occurred on an average of 2.5 times more frequently than transversions (Lemey et al., 2009). Our result shows that there is no significant difference for substitution tendency among the four transitions (**Table 2**). At the transition or transversion sites, the average ratio of each substitution varied without clear preferences, e.g., the average ratio of A → T transversion (2.79%) was much higher than A → G transition (0.80%). The average ratio of C → A transversion (2.12%) was similar to C → T transition (2.19%; **Table 2**).

**Table 2:** Substitution tendency among RSV quasispecies in different hosts.

Substitution type		Substitution number		Substitution ratio	
		*O. sativa*	*N. benthamiana*	*O. sativa*	*N. benthamiana*
A→	G	61	345	0.80%	7.67%
	C	16	34	0.24%	2.63%
	T	16	90	2.79%	8.11%

T→	C	68	353	1.56%	7.69%
	A	21	90	0.21%	6.54%
	G	9	36	0.21%	7.08%

G→	A	58	326	1.97%	5.57%
	C	3	2	0.08%	2.62%
	T	18	40	0.77%	6.42%

C→	T	51	299	2.19%	7.84%
	A	16	57	2.12%	6.87%
	G	4	3	0.35%	3.52%

**FIGURE 4:**
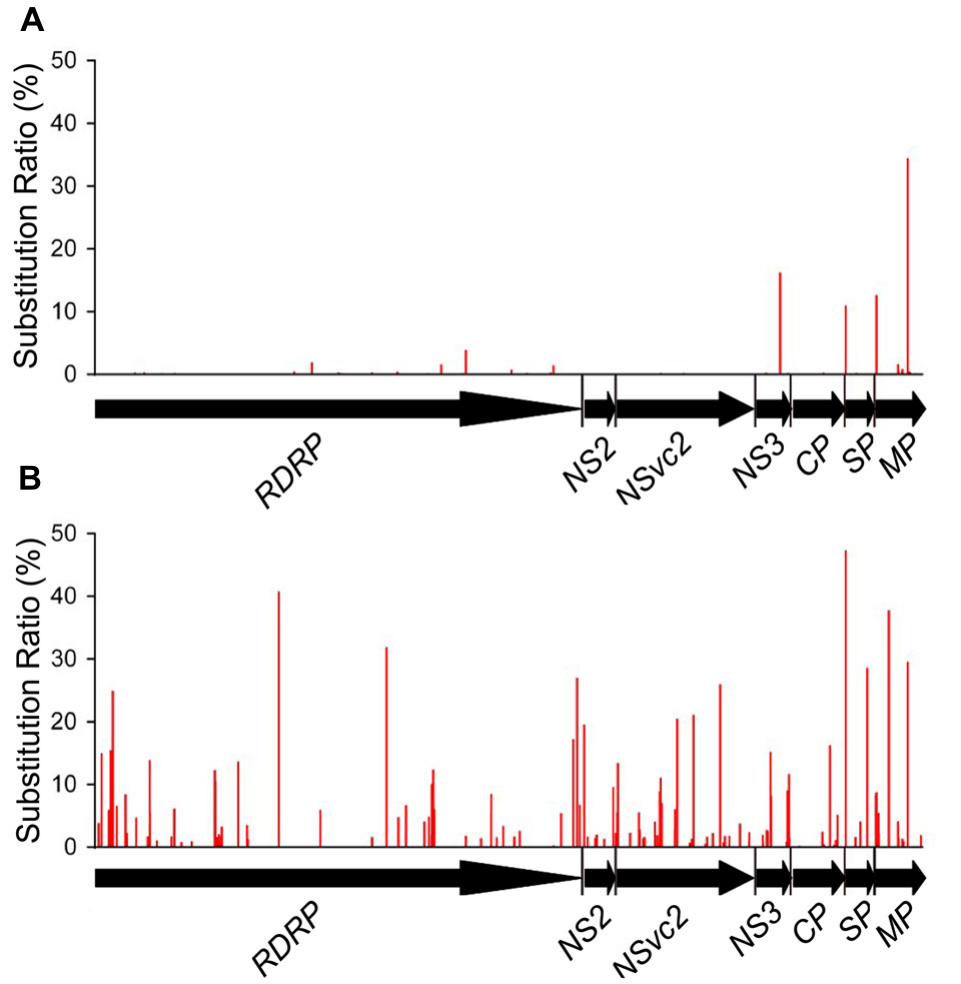
**Non-synonymous substitution patterns in RSV ORFs from infected *O. sativa* (A) and *N. benthamiana***(B)** plants**. The seven RSV ORFs are shown by horizontal arrows, and the vertical coordinates represent the ratios of substitutions derived from the master sequence of each populations at each site (number of substituted nucleotide observed/number of total nucleotide sequenced).

Non-synonymous substitutions were also found in 71 codons located within 6 of the 7 ORFs analyzed. These include 25 codons in *RdRp*, 7 in *NSvc2*, 9 in *NS3*, 12 in *CP*, 8 in *SP*, and 10 in *MP*. No non-synonymous substitution was detected in *NS2* ORF. Only 4 out of the 71 codons had substitution ratio above 10%. These include the 150th codon in the *NS3* ORF (16.19%, Pro → Leu), the 8th codon in the *SP* ORF (10.92%, Val → Ile), and the 12th and the 200th codons in the *MP* ORF (12.62 and 34.45%, Ser → Asn and Ile → Val, respectively; **Figure 4A**). The sites of synonymous substitutions were 3.5 times higher than those of non-synonymous substitutions. The average substitution ratio of synonymous substitution was 1.64 times higher than that of non-synonymous substitutions.

### Genetic Diversity of RSV Populations in *N*. *benthamiana*

To investigate the role of host in RSV evolution, the virus was mechanically inoculated in *N*. *benthamiana*, an experimental host of RSV. In infected *N. benthamiana* plant, substitutions were found at 1675 sites in the RSV populations, approximately five times more than those found in the RSV populations from infected *O. sativa* plant. The average nucleotide diversity and substitution ratio were also very different between these two groups of RSV populations. For RSV populations from *N. benthamiana*, substitutions were detected in 874 sites in RNA 1 (9.74%), 368 sites in RNA 2 (10.47%), 204 sites in RNA 3 (8.24%), and 149 sites in RNA 4 (6.91%). The substitutions occurred more densely in RNA 1 and 2. There were 206 substitution sites with the substitution ratios above 20%, and more than half of them were found in RNA 1 (**Figure 2B**).

Our results also indicate that the average nucleotide diversity in each ORF and non-coding regions is higher for RSV populations from *N. benthamiana*, especially in the *RdRp*, *NS2*, and *NSvc2* ORFs and the non-coding region. Consequently the differences of average nucleotide diversities among the ORFs and non-coding regions are decreased. The highest average nucleotide diversity is in the *NSvc2* ORF (12.97%) and the lowest average nucleotide diversity is in non-coding regions (6.14%; **Figure 3A**). The average substitution ratios are also increased in all ORFs and non-coding regions analyzed. The highest average substitution ratio is found in the *SP* ORF (9.25%) and the lowest in the *CP* ORF (3.75%). Interestingly, the average substitution ratio is not increased in non-coding regions (**Figure 3B**).

A total of 1323 transition sites and 352 transversion sites were detected in the sequences of RSV populations from *N. benthamiana*. The transitions occurred on an average of 3.76 times more frequently than the transversions, but no significant difference in frequency was observed for the four transition groups (**Table 2**). The average substitution ratios of the four transition groups varied without a clear preference. The highest average substitution ratio was for transversion A → T (8.11%) found in RSV populations from* O. sativa* (**Table 2**).

Non-synonymous substitutions were found in 147 codons distributed in the seven ORFs. These include 60 non-synonymous substituted codons in *RdRp*, 6 in *NS2*, 35 in *NSvc2*, 16 in *NS3*, 11 in *CP*, 6 in *SP*, and 13 in *MP* ORF. Twenty-four of the 147 codons had substitution ratios above 10%, and two of them were detected at the eighth codon in the *SP* ORF and the 200th codon in the *MP* ORF, respectively. It is worth to mention that the RSV populations from *O. sativa* also had high substitution ratios at these two codons. There are 24 unique non-synonymous substitutions in the RSV populations from *N. benthamiana*, likely to be the mutations caused by the interaction between RSV and *N. benthamiana* (**Figure 4B**). The average substitution ratios of the synonymous and non-synonymous substitutions were about five times of those found in the RSV populations from *O. sativa*.

### Population Structures of RSV in Different Host Plants

The master sequences of RSV populations from the infected *O. sativa* and *N. benthamiana* plants were identified after mapping the reads from the high-throughput sequencing data on the reference sequences of RSV genome segments. There are 156 different (0.9% of genome nucleotides) substitutions between the two master sequences. The master sequence from infected *O. sativa* plant is composed of 39.09% GC nucleotides while that from the infected *N. benthamiana* plant is 38.92%. When the substitution tendency bias was analyzed, more transitions than transversions (approximately fourfold) were observed, and there were remarkably more G/C to A/T substitutions than the reverse substitutions (**Table 3**). After RSV was transmitted from *O. sativa* to *N. benthamiana*, the G/C ↔ A/T substitutions varied among the regions in the RSV genome (**Table 4**). For example the G/C ↔ A/T substitutions were similar in the non-coding regions of the two master sequences. In the coding regions, the G/C → A/T substitutions seemed to occur more often than the reverse substitutions (**Table 4**). As reported previously, the rice genome is GC rich and rice coding sequences contain 45–75% G and C, whereas the tobacco genome is GC poor and its coding sequences contain 40–60% G and C (Salinas et al., 1988; Matassi et al., 1989; Carels and Bernardi, 2000). The decreased GC level after the virus was transmitted from *O. sativa* to *N. benthamiana* suggests that RSV may adjust its codon usage to ensure its efficient protein translation in a new host.

**Table 3:** Substitution tendency of the master sequence after RSV was transmitted from *O. sativa* to *N. benthamiana.*

Original base	No. of substitutions at mutated base				
	A	C	G	T
A	–	2	27	11
C	3	–	0	45
G	34	0	–	1
T	7	21	5	–

**Table 4:** Number of A/T ↔ G/C substitutions in the master sequence after RSV was transmitted from *O. sativa* to *N. benthamiana.*

Transition type	Transition number							
	Non-coding	*RdRp*	*NS2*	*NSvc2*	*NS3*	*CP*	*SP*	*MP*
A/T → G/C	14	26	1	4	4	1	1	4
G/C → A/T	14	45	2	12	3	4	3	1

## Discussion

Since the first introduction of quasispecies hypothesis by Eigen and Schuster (1977), this population-based framework has been used extensively to study RNA virus evolution. Based on the complete genomic sequences or partial genomic sequences of RSV isolates, RSV isolates in China were mainly divided into two subtypes: subtype II from Yunnan province and subtype I from rest parts of China, RSV isolates in subtype II were more genetically diverse than those in subtype I, and the mean genetic distance of RSV genes between two subtypes, within subtype I and subtype II ranged from 0.0529 to 0.0865, 0.0123 to 0.0256, and 0.0183 to 0.0387, respectively (Wei et al., 2009; Huang et al., 2013). In this study, we used the high-throughput sequencing technology to analyze and profile RSV populations from infected *O. sativa* or *N. benthamiana* plants. Two parameters, average nucleotide diversity and average substitution ratio at single sites, were obtained through high-throughput sequencing and used to evaluate genetic diversities in RSV populations from an infected *O. sativa* or *N. benthamiana* plant. For the whole RSV genome, the average nucleotide diversity and average substitution ratio were 1.99% and 1.47% from *O. sativa*, and 9.79% and 7.05% from *N. benthamiana*, respectively. RSV isolates in China were reported to have low genetic diversity because of its narrow host range (Wei et al., 2009). Our study find the diversity of RSV populations increased after RSV was transmitted from *O. sativa* to *N. benthamiana*. *N. benthamiana* might provide quite different select pressure from *O. sativa*, and this observation also gave a direct evidence that virus might allow higher genetic diversity for host adaptation (Fargette et al., 2006).

The two parameters are independent of each other regardless of the origin of the two virus populations. For example, the average nucleotide diversity in the *CP* ORF was the highest in the RSV populations from the infected *O. sativa* plant, whereas the average substitution ratio in this ORF was much lower than some of the other ORFs (**Figure 3**). Also transitions were much higher than transversions in the RSV populations from both infected *O. sativa* and *N. benthamiana* plants (**Table 4**), but the average substitution ratio did not have difference (**Figure 2**). These results indicate that these two parameters are likely driven by different factors.

The genetic diversity in RNA virus populations was considered to be controlled by the interactions between host and viral factors (Schneider and Roossinck, 2001). So there were at least two steps during RNA virus evolution: substitutions emerged via replication errors and the selection of the original and substituted bases by host. During the second step, the accumulation of the less adaptive one might be repressed by host–virus interaction. So if host–virus interaction is in favor of the substituted base, it will accumulate more than the original base and eventually replace the original one. Otherwise, the substituted base will accumulate at lower level or even be eliminated. So substitution ratio reveals the destiny of each substitutions under host selection. Thus the sites with high substitution ratios may be essential in virus evolution in corresponding host. In this study, sites with high substitution ratio in *O. sativa* and *N. benthamiana* were documented. Further work is necessary to confirm their function in virus evolution and host–virus interaction.

The observation that the average substitution ratio of non-coding regions was not changed after RSV was transmitted form *O. sativa* to *N. benthamiana* indicates that the RSV non-coding sequences may not play an important role in RSV–host plant interactions. On the other hand both synonymous and non-synonymous substitution ratios were remarkably increased after RSV was transmitted form *O. sativa* to *N. benthamiana*. This observation suggests that both synonymous and non-synonymous substitutions are also essential in RSV–host plant interactions. Possible scenarios that may be used to explain our hypothesis are that the non-synonymous substitutions may alter features of RSV protein(s) to better interact with specific host factors during its infection, e.g., replication and movement in plant, while synonymous substitutions alter the codon composition to accord with host codon usage bias.

## Conclusion

We find the RSV populations from infected *N. benthamiana* plant is more diverse than that obtained from rice and the high-throughput sequencing technology is a powerful technology to investigate population genetic diversity of plant RNA viruses.

## Conflict of Interest Statement

The authors declare that the research was conducted in the absence of any commercial or financial relationships that could be construed as a potential conflict of interest.
